# Editorial: Microbial degradation of agricultural waste

**DOI:** 10.3389/fmicb.2026.1856978

**Published:** 2026-05-13

**Authors:** Nadeem Tahir, Tünde Pusztahelyi

**Affiliations:** 1 College of Mechanical and Electrical Engineering, Henan Agricultural University, Zhengzhou, China; 2 Central Laboratory of Agricultural and Food Products, Faculty of Agricultural and Food Sciences and Environmental Management, University of Debrecen, Debrecen, Hungary

**Keywords:** agricultural, biotechnology, microbiome, valorization, waste management

The sustainable valorization of agricultural biomass and organic residues is integral to the advancement of circular bioeconomy systems and climate-resilient agricultural practices. Vast quantities of lignocellulosic residues, livestock manure, and agro-industrial by-products are generated annually worldwide, presenting both significant environmental challenges and substantial opportunities for resource recovery. Their efficient conversion into renewable energy, value-added bioproducts, and sustainable agricultural inputs is therefore essential for reducing waste accumulation, lowering greenhouse gas emissions, and improving resource-use efficiency across agroecosystems. The studies assembled in this Research Topic collectively highlight recent progress in microbial biotechnology, enzymatic catalysis, and process engineering that is advancing the biological conversion of agricultural residues toward more sustainable and higher-value applications.

A central theme emerging from this Research Topic is the optimization of composting and organic waste recycling through targeted biological and process-based interventions, particularly the application of microbial inoculants and bioactive amendments. While composting remains a cornerstone of agricultural waste management, its performance is often limited by carbon loss, greenhouse gas emissions, and incomplete humification. In this context, Liu, He et al. demonstrate that the combined application of biochar and cellulase during co-composting of cattle manure and sugarcane bagasse can simultaneously mitigate CO_2_ and CH_4_ emissions while promoting the formation of humic substances. Their findings further reveal that this synergistic treatment reshapes microbial succession by enriching lignocellulose-degrading and humification-associated taxa while suppressing microorganisms linked to greenhouse gas production. These results exemplify how microbiome-informed amendment strategies can improve both the environmental footprint and agronomic value of composting systems.

The importance of microbial inoculants in enhancing lignocellulosic decomposition is further underscored by studies identifying key functional taxa associated with biomass turnover during composting. In particular, the isolation of multifunctional strains such as *Bacillus tequilensis* JZF3 capable of lignocellulose degradation, pathogen suppression, and plant growth promotion highlights the emerging potential of integrated bioaugmentation approaches that couple residue decomposition with crop health benefits (Liu, Liu et al.). Such multifunctionality may support the development of next-generation microbial products with broader agricultural utility beyond waste treatment alone.

Progress in inoculant formulation is likewise reflected in the development of biodegradable multilayer biocapsules for sustained microbial delivery (Senadheera et al.). By improving inoculant protection, persistence, and controlled release, these encapsulation systems enhanced thermophilic composting duration, nutrient retention, and compost maturity, demonstrating the value of integrating biomaterials engineering with microbial process optimization. Together, these studies indicate that inoculant performance depends not only on microbial function but also on delivery strategy and environmental compatibility.

Beyond controlled composting systems, several contributions address the challenges of biomass decomposition under field conditions. In cold and arid agricultural soils, microbial inoculants combined with optimized straw-returning practices significantly accelerated maize straw degradation through coordinated shifts in bacterial community structure and extracellular enzyme activity (Zhao et al.). These findings emphasize the importance of considering agronomic context when deploying microbial technologies and demonstrate that successful biomass valorization *in situ* requires alignment between microbial function and environmental management.

Renewable bioenergy production from lignocellulosic biomass constitutes another major focus of this Research Topic. Usman et al. provide a comprehensive overview of nanotechnology enabled photo-fermentative hydrogen production, highlighting the capacity of nanophotocatalysts to enhance biomass depolymerization, electron transfer, and photobiological hydrogen generation. By incorporating technology readiness, techno-economic, and life cycle perspectives, the authors provide a valuable translational framework for assessing the future scalability of nano-enabled hydrogen production systems. Their review illustrates the growing convergence of microbial biotechnology, materials science, and reactor engineering in the pursuit of sustainable bioenergy platforms.

Complementing these hydrogen-focused approaches, new insights into anaerobic methane generation are presented through the GET rice straw biogas production system, in which *Bacillus fumarioli* was identified as a dominant microbial contributor associated with enhanced methane production (Chen et al.). This observation suggests that previously underappreciated taxa may play important roles in anaerobic lignocellulose conversion and methanogenic consortia, broadening current understanding of microbial ecology in biogas-producing systems.

Enzymatic biomass depolymerization continues to expand the scope of agricultural residue valorization. Wang et al. report a highly efficient chitinase system capable of directly hydrolyzing powdered chitin without acid pretreatment, representing a meaningful advance toward greener processing of crustacean shell waste into glucosamine and chitooligosaccharides. Similarly, the enzyme-based production of xylo- and malto-oligosaccharides from cereal biomass, reviewed by Kostanova et al., illustrates the increasing diversification of biomass valorization pathways toward high-value functional ingredients. Together, these studies reinforce the concept that agricultural residues can serve not only as substrates for bulk composting and energy generation but also as feedstocks for speciality biochemical production.

Additional contributions further extend the application range of microbial biomass valorization into biodegradable material degradation (Kim et al.), mitigation of antibiotic resistance genes during manure composting (Jin et al.), rhizosphere engineering (Zhang et al.), feed production from waste materials (Liu, Li et al.), and peat-free horticultural substrate development (Shi et al.). These studies collectively highlight the broader ecological and agronomic relevance of microbial and enzymatic interventions across agricultural production systems.

Taken together, the studies in this Research Topic reflect a broader shift in biomass valorization research from empirical process optimization toward mechanistically informed biological engineering. The increasing integration of high-throughput sequencing, metagenomics, metabolomics, and ecological network analysis is enabling a deeper understanding of microbial community assembly, functional succession, and metabolic interactions during biomass transformation. Such advances provide the mechanistic basis for rationally designing targeted strategies to improve conversion efficiency, product quality, and environmental sustainability.

Despite this progress, several challenges remain before many of these technologies can achieve widespread practical implementation. Inoculant stability, environmental robustness, catalyst cost, biosafety considerations, reactor design limitations, and economic feasibility continue to constrain scale-up and commercialization. Addressing these barriers will require sustained interdisciplinary collaboration across microbial ecology, enzyme engineering, agronomy, biomaterials science, and process systems engineering.

In summary, the contributions to this Research Topic underscore the expanding potential of microbial and enzymatic technologies to transform agricultural biomass into renewable energy, sustainable agricultural inputs, and high-value bioproducts. By advancing both mechanistic understanding and applied innovation, these studies provide an important foundation for the continued development of efficient, scalable, and sustainable biomass valorization strategies in support of future circular bioeconomy systems.

